# Acute malnutrition and food insecurity in Yemen, 2021: Evidence from a two-stage cluster randomised survey in a protracted crisis

**DOI:** 10.1371/journal.pgph.0004331

**Published:** 2025-07-11

**Authors:** Mariana Perez Duque, Abdulwahab Alburhomy, Ali Ahmed, Yousef Saleh, Anja Wolz, Ana B. Abecasis, Neil J. Saad Duque, Henrik Salje, Julita Gil-Cuesta, Jo Robays, Raphael Veicht, Saleem Alazab

**Affiliations:** 1 Médecins Sans Frontières, Aden, Yemen; 2 Pathogen Dynamics Unit, Department of Genetics, University of Cambridge, Cambridge, United Kingdom; 3 Global Health and Tropical Medicine, Associate Laboratory in Translation and Innovation Towards Global Health, LA-REAL, Institute of Hygiene and Tropical Medicine, NOVA University of Lisbon (IHMT-UNL), Lisbon, Portugal; 4 Johns Hopkins Center for Humanitarian Health, Baltimore, Maryland, United States of America; 5 Médecins Sans Frontières, Luxembourg Operational Research Unit (LuxOR) Luxembourg, Luxembourg, Luxembourg; 6 Médecins Sans Frontières, Operational Centre Brussels, Medical Department, Ixelles, Belgium; St John's Medical College, INDIA

## Abstract

The ongoing conflict in Yemen, which began in 2014, has led to one of the world’s most severe humanitarian crises. The Hudaydah region, located on the Red Sea coast and home to the country’s second-largest port, is critical for the delivery of food and medical supplies. We conducted a two-stage cluster randomised survey to estimate the prevalence of acute malnutrition among children and pregnant and lactating women (PLW). We estimated the prevalence of household food insecurity and quantified death rates. During February-March 2021, acute malnutrition prevalence was 14.1%(95%CI: 12.2-16.2) among children aged 6–59 months, with 4.0%(95%CI: 3.3-4.9) severely malnourished. 43% of malnourished children were not in a nutritional programme. Acute malnutrition among PLW was 25.7%(95%CI: 23.0-28.6). We estimated 54%(95%CI: 44–63) of households were food insecure, 22%(95%CI: 15–31) severely. Crude and under-five death rates were below humanitarian thresholds. More than half of the children reported sickness in the last 14 days, and this proportion was higher among the malnourished.

## Introduction

Acute malnutrition among children is a severe health condition that can be life threatening, and negatively impacts long-term health and development [1,2]. Malnutrition translates to the deficit, excess or imbalance of energy intake and/or nutrients. It includes two groups of conditions: undernutrition and micronutrient malnutrition and overweight. Undernutrition includes wasting (low weight-for-height), stunting (low height-for-age) and underweight (low weight-for-age). Currently, more than 45 million children suffer from wasting worldwide as a result of social, economic and environmental factors. More than three quarters of severely wasted children live in Asia, including the Arabian Peninsula in Western Asia. In low-middle income countries the primary cause of wasting is related to inadequate food access [3]. Yemen, located on the Arabian Peninsula, has for several years been one of the world’s most severe humanitarian crises. Currently, the World Bank estimates that more than two thirds of the 30 million population in Yemen is in need of acute assistance [4].

The Hudaydah region in Yemen has the second largest port in the country, which receives about 80% of all humanitarian supplies. Therefore, it is of strategic interest and this region experienced one of the most important active frontlines of the ongoing conflict, which started in 2014. Due to security reasons, limited access in Hudaydah prevented suitable evidence collection to assess humanitarian and medical needs [5]. Internally displaced persons (IDPs) in Yemen remain one of the most vulnerable groups due to the ongoing conflict and the resulting humanitarian crisis. In 2021, over 4 million people were internally displaced, with 119,364 newly displaced individuals recorded during the year [6]. In October 2020, during a period of active conflict, a prevalence of 27% of global acute malnutrition (GAM) was estimated among children under five years of age and 31% prevalence among pregnant and lactating women (PLW) living in the Southern Hudaydah region [7]. Evidence collected in limited access areas frequently uses *ad hoc* methods, such as non-representative sampling, non-conventional target groups, or different time periods for classification [8,9]. Due to the lack of recent survey data, concerns about data quality, and medium reliability, the levels of acute malnutrition and food insecurity in Yemen were likely underestimated. To determine whether current interventions were sufficient to address the emergency and, if necessary, design a tailored response, an urgent need arose for a representative survey focusing on the most vulnerable groups in one of the regions most affected by the conflict.

Rapid nutrition assessments are common in emergencies where it is critical to measure acute malnutrition in a timely manner. The most widely accepted practice is to assess malnutrition levels in children aged 6–59 months as a proxy for the entire population [10]. Also, given their additional nutritional needs, PLW are also at a higher risk of malnutrition than other groups in the population. Anthropometric measurements combined with clinical assessment are frequently the method used to assess the nutritional status of individuals in emergency settings. According to these measurements, GAM can be divided into moderate acute malnutrition (MAM) and severe acute malnutrition (SAM) [11]. Malnutrition cases require medical care, such as targeted supplementary feeding programmes, therapeutic feeding for severely malnourished children and treatment of acute medical conditions. Supplementary and therapeutic feeding programs are critical strategies in addressing malnutrition, particularly in emergency contexts. Supplementary feeding targets individuals at risk of malnutrition, providing additional food to bridge nutritional gaps, often in the form of fortified products. Therapeutic feeding, on the other hand, is a lifesaving intervention for individuals, especially children, suffering from severe acute malnutrition. It involves intensive care using energy-dense therapeutic foods and medical treatment to promote recovery. Both programs are essential components of broader public health efforts, aiming to reduce mortality and improve nutritional outcomes among vulnerable populations [12]. Because malnutrition is a life-threatening condition, crude and age-specific death rates at the scale of the crisis-affected area are crucial to support humanitarian intervention. Crude death rates (CDR) give a broad picture of overall mortality, reflecting the general severity of a crisis, while age-specific death rates highlight vulnerabilities within a particular demographic group, such as children. These indicators help identify populations at greatest risk, enabling humanitarian organisations to prioritise interventions such as nutrition, healthcare, and shelter in areas of highest need [13]. Estimates on death rates in the Southern Hudaydah region were limited, and population needs were not obvious, especially due to scarce communication and difficult access. Most available data focused on the overall direct and indirect deaths in the country and deaths taking place outside health facilities and in the communities in this region were unknown.

The United Nations (UN) Food and Agriculture Organization (FAO) defines food insecurity as a situation where there is a lack of sufficient quantity and quality of food, therefore people do not have enough to develop, grow and have a healthy life. Thus, food insecurity is a major driver of malnutrition, impacting dietary quality and quantity. Food-insecure households are more likely to experience inadequate caloric intake and poor dietary diversity, both of which contribute to malnutrition, particularly in vulnerable groups such as children and pregnant women [14]. Major drivers of food insecurity and malnutrition include conflict, extreme climate events, economic downturns, and inequality, have been highlighted in recent reports leading the world off track to meet the Sustainable Development Goals (target 2.1 and 2.2) [15]. In conflict-affected areas, food insecurity is exacerbated by disruptions in food production, market accessibility, and distribution systems, further contributing to malnutrition [16]. Food insecurity acts as both a direct and indirect contributor to malnutrition, impacting individuals’ ability to access and utilize nutritious food and increasing their risk of illness [17].

With the aim to better understand the health and nutritional situation of the population, identify groups in greatest need for nutritional support and guide the overall Médecins Sans Frontières (MSF) medical humanitarian strategy in the Southern Hudaydah region, this study aimed to estimate the prevalence of acute malnutrition, food insecurity and death rates along one of the most severely affected regions in Yemen.

## Methods

### Study setting

The data collection was conducted between February 28 and March 15, 2021, in five Southern Hudaydah districts (Khawkhah, Durayhimi, Bayt Al Faqih, Tuhaytah, and Haiz) with a population of 236,011, including 48,754 children under five years of age (District Health Authorities shared data). The assessment was coordinated by MSF with the collaboration of the Ministry of Public Health and Population and District Health Authorities.

### Study design and participants

We conducted a cross-sectional regionally representative study, focused on the effects of the protracted conflict on health, food security, and mortality in the Southern Hudaydah region. We used a probabilistic, two-stage, clustered sampling strategy (S1 File). Access to this region for humanitarian assistance was highly restricted due to the conflict. To overcome these challenges, MSF, in collaboration with District Health Authorities, developed a geo-mapped nominal list detailing the estimated population numbers of all villages and IDP camps. Based on sample size calculations (S1 File), a sampling plan of 40 clusters was surveyed to obtain the total number of 1480 households, with each cluster containing 37 households. We used SMART software to randomly assign clusters, with the chance of each cluster (village) being chosen proportional to its population size [18]. This technique allowed every village the same probability of being selected. Eligible participants for this assessment included all individuals living, for at least one month (including children born), in the randomly selected household, during the period of the assessment. Inclusion criteria for each participant included living in the household at the time of the study and giving informed, free, and oral consent, provided by the head of the household. Exclusion criteria included refusal to participate in the survey; cluster villages situated in direct proximity to an active frontline; cluster villages abandoned by the population; absence of the head of the household or an adult capable of consenting. To measure malnutrition in the population we assessed children aged between 6 and 59 months and PLW. All households were assessed for basic demographic information, including food insecurity.

### Procedures

Study interviewers administered structured questionnaires to all participants, through the head of the household or adult, to capture demographics, household size, and health status of each household member. Interviewers received training prior to data collection to ensure standardisation of methods and a one-day pilot. was conducted in each district (district interviewers). An electronic standardised questionnaire using Kobo Collect tool mobile (version 1.14.0a) was used for data entry [19]. Data was downloaded daily upon return from each sampling site. Villages in Southern Hudaydah were considered as the smallest geographical unit (clusters). The household was considered as the basic sampling unit. A household was defined according to the local context as all individuals, whether related or not, who usually live in the same dwelling, at least for the last month, shared a daily meal in this household, jointly manage all or part of their resources, and recognize the authority of a single person called the head of the household. The head of the household was defined as an adult member of the household who was at least 15 years of age, and could provide accurate information about their household, including describing with reasonable accuracy the events that occurred during the recall period, lived in the household for the entire or majority of the recall period, and was present at the time of the study. A current household member was defined as an individual who was a member of the household according to the household definition and who was present at the time of the study, or who was present in the household the previous night. All malnourished children and PLW identified during the survey who were not already enrolled in a nutritional program were referred to the nearest health facility, along with those currently in a nutritional program who were found to have new acute medical conditions at the time of the survey. Prior to the surveys, MSF collaborated with the District Health Authorities to identify functional health centers for admission and care. Additionally, all participants’ households were provided with soap, with the quantity distributed corresponding to the number of children under 5 years of age.

### Variables and data

We collected household- and individual-level data using a questionnaire as described. Household level data included the number of individuals living in the household at the time of the assessment, and Food Insecurity Experience Scale (FIES) questions. Internal displacement status was determined based on self-reported information from households regarding their displacement history, including whether they had been forced to leave their place of origin due to conflict, natural disasters, or other crises. The household’s food insecurity was assessed by the FIES [20,21], an international comparable tool that measures the severity of food insecurity based on self-reported experiences to eight binary response (yes/no) questions (S2 File). These questions were ordered by an increasing severity experiencing scale. Individual level data included age, sex, left and/or new in the household during the recall period, birth during the recall period, death during the recall period and, if death, date and place of death, cause of death if known, healthcare-seeking behaviour. The recall period chosen was three months (90 days) prior to the interview day. Additionally, for children aged under-five years we asked about self-reported illness in the last 14 days, healthcare-seeking behaviour, and clinical syndrome. For children from 6 to 59 months and PLW we measured the Mid-Upper Arm Circumference (MUAC) and clinically assessed the presence of bilateral oedema (children). We questioned PLW about the gestational age of pregnancy. For both children and PLW we asked if they were currently or previously enrolled in therapeutic or supplementary nutritional programs. MUAC was systematically measured by the circumference of the arm at mid-point to the nearest millimetre (mm), in children from six to 59 months of age and among PLWs using a non-stretch graduated tape, following the SPHERE guidelines [10], MUAC cutpoints below 115mm and 185mm to be used for identifying and targeting children between 6–59 months and PLW with SAM, respectively. For MAM, intervals for MUAC measures were between 115–125mm among children between 6–59 months and between 185–230mm for PLW. Additionally, a child presenting bilateral pitting oedema was categorised as having SAM, irrespective of MUAC measurements [10].

### Statistical analysis

Demographic, clinical (including morbidity), and nutritional data were summarised using frequencies and proportions for categorical variables and median and interquartile for continuous variables. Prevalence of acute malnutrition was calculated directly by ENA Smart software considering all parameters of the calculated sample sizes (S1 File). Nutrition analyses were stratified by age groups and sex. For groups’ comparison, chi-squared tests and logistic regression models were used to analyse the association between the nutritional status and demographic and health characteristics and calculate odds ratios (ORs) and corresponding 95% confidence intervals (95%CI). Differences in proportions were measured using the Pearson test χ2 and p-value values are presented. We also compared individual MUAC measurements to existing age- and sex-specific estimates from non-malnourished populations in 22 different Lower- and Middle-Income Countries (LMIC) [22]. We used a logistic regression to identify individual level factors associated with GAM. We modelled age, sex and reported sickness in the last 14 days as fixed effects. We assessed multicollinearity among covariates by calculating the variance inflation factor (VIF) for each one. We used a VIF threshold of 5 to assess collinearity among covariates, with no model adaptation necessary for values below this threshold. To account for possible heterogeneity among clusters (villages) we added the sampling cluster as a random effect. We combined both fixed and random effects (mixed effects) in a logistic regression model within a Bayesian framework of generalised linear models. To estimate the parameters, we used the Integrated Nested Laplace Approximation (INLA) method [23]. To test how much cluster-specific information would benefit the model we calculated the intraclass correlation coefficient (ICC). We initially took all covariates one-by-one in univariate analysis. We then identified all covariates that were significant at the 0.05 p-value level for inclusion in the multivariable model.

We estimated death rates retrospectively. The crude death rate (CDR) was defined as the number of people among all study participants who died over the recall period, over the total study population in the recall period [13]. The zero-to-five death rate (0–5DR) was defined as the number of deaths among children from zero to five years of age among total study participants from zero to five years of age, expressed as deaths per 10,000 per day (S3 File).

The FAO recommends monitoring food insecurity using the Food Insecurity Experience Scale (FIES), a standardised tool to provide internationally comparable estimates of food insecurity in terms of the experiences towards access to food [21]. The FIES produces estimates in two levels of the severity of food insecurity, and uses a statistical framework that calibrates the answers of eight food access questions against a standard reference scale [20]. The FIES data analyses used item response theory by applying the Rasch model framework [20]. The analysis involved parameter estimation: calculation of the severity of food insecurity associated with each assessment question (item) and each household; statistical validation: the assessment of whether, depending on the quality of the data collected, the measure is valid based on measure-of-fit tests (infit); calculation of measures of food insecurity: household probabilities, for each sampled household the probability of the household experiencing food insecurity above a given level of severity is calculated, based on their responses to the FIES items, and population prevalence estimates, to estimate the prevalence of food insecurity at moderate and severe levels in the population. Participants with a missing response to any of the eight FIES items were excluded from the Rasch analysis. All analyses were conducted using R software version 4.2.1 and figures were produced using the *ggplot2* package version 3.4.4 [24].

### Ethical considerations

This survey was approved by the Ministry of Public Health and Population in Aden (A21/4997). This research fulfilled the exemption criteria set by the Médecins Sans Frontières Ethics Review Board (ERB) for a posteriori analysis of routinely collected clinical data and thus did not require MSF ERB review. It was conducted with permission from the Medical Director, Operational Center Brussels, Médecins Sans Frontières. Survey objectives were shared with community representatives and local authorities, ahead of the survey and dates of the survey were jointly agreed. These community interactions enhanced safety and security of the study team and participants and improved the survey. Given the challenges of working near the frontlines of the war where our target population lived, we anticipated risks such as security threats, breaches of confidentiality, and psychological discomfort, and implemented mitigation measures accordingly.

Free voluntary and informed verbal consent was obtained from all participants, including from the head of household, adults and children’s parents or guardians before conducting the survey, with the right to refuse or withdraw. The survey was explained and conducted in the local language (Arabic) by local native speakers. The informed consent process was documented using the survey electronic questionnaire and approved by the MoH and in accordance with the MSF protocol. The interviewer explained the contents of the consent form and clearly stated the participant’s right to refuse or accept participation in the survey or to withdraw at any point. Upon the participant’s declaration, the interviewer recorded the date, time, and status of the consent as either “approved” (if the participant agreed and completed the interview) or “rejected” (if the participant declined to participate or withdrew before completion). Rejected participations were excluded from the dataset and analysis. As this was a verbal consent process, the interviewers served as a witness to the participant’s consent.

All children and PLW with MUAC measures below the cut-off points during the assessment and who were not yet being followed in a nutritional program or showed signs and/or symptoms of acute disease were referred to the nearest health facility for medical care. A referral form was given to the head of household or an adult to facilitate care upon their arrival in the health facilities. Before the study began, MSF identified, together with the District Health Authorities, the functional healthcare centres for children admission and care. To protect confidentiality of the participants’ data, electronic data collection tools were used in offline mode, with the global position system disabled. Data were downloaded daily and securely stored in a password-protected system, accessible only to authorised survey team members.

### Role of funding source

Médecins Sans Frontières (MSF) provided support in the form of salaries for MPD, Ab.A, AA, YS, AW, JGC, JR, RV, SA. MSF programmatic funding covered all costs associated with the survey which was conducted for operational purposes. MSF was involved in the study design, data collection and analysis, decision to publish, and preparation of the manuscript. AA is funded through funds from Fundação para a Ciência e Tecnologia (FCT) to GHTM-UID/04413/2020 and LA-REAL-LA/P/0117/2020. MPD is currently funded by Gates Cambridge Trust (OPP1144), which had no role in the study and decision to publish.

### Inclusivity in global research

Additional information regarding the ethical, cultural, and scientific considerations specific to inclusivity in global research is included in the Supporting Information (S1 Checklist).

## Results

**Fig 1:**
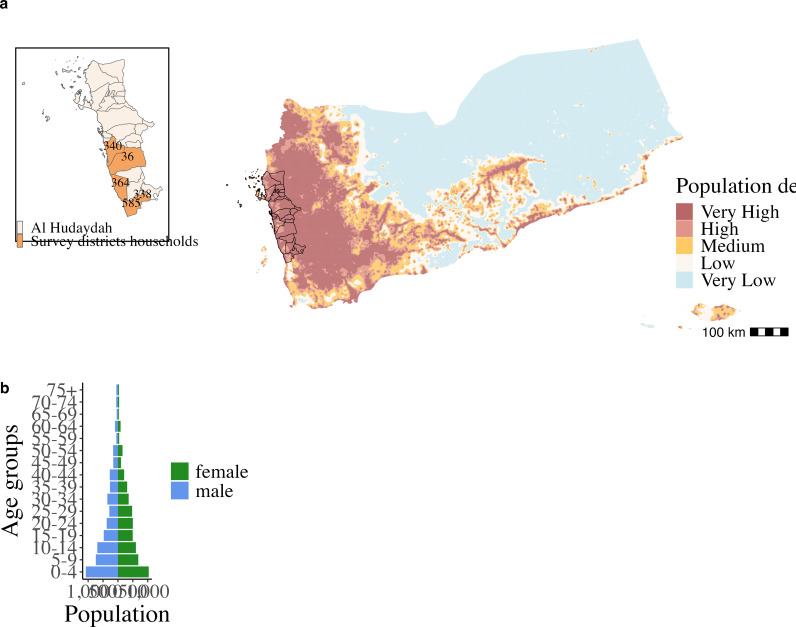
Study setting and population. (A) Yemen population density in a logarithmic relative scale. Al Hudaydah region bordered black. Inset represents Al Hudaydah district’s borders, with Al Khawkhah, Al Durayhimi, Bayt Al Faqih, Al Tuhaytah and Haiz survey districts in orange colour. Text labels inside districts correspond to the number of households surveyed in each district (sampled proportional to size). To obtain the population size in each cell, we used the WorldPop data on population density by Km^2^ [49]. (B) Age distribution of the study population in 5-year categories, grouped by sex (female - green, male - blue). Base map credit: Country and administrative levels boundaries are provided under an open license (CC-BY) by GADM, v.4.1 [50]. Yemen population density raster file is provided under an open licence (CC-A) by WorldPop [49].

Between February 28 and March 15, 2021, a total of 10,360 individuals from 1,657 households (Table 1) in 40 randomly selected communities in five districts (Khawkhah, Durayhimi, Bayt Al Faqih, Tuhaytah, and Haiz) of the Southern Hudaydah region, in Yemen Red Sea coast (Fig 1A), participated in the survey. Median household size was six members (interquartile range (IQR) 4–8). Half of the population surveyed was female (Fig 1B), with a median age of 16 years (IQR 6–30). Internally displaced people represented 35.8% of the surveyed households. Individuals with 35.2% households from Khawkhah, 22.6% from Durayhimi and Bayt Al Faqih districts, 21.9% from Tuhaytah and 20.3% from Haiz.

**Table 1:** Household and individual characteristics, Southern Hudaydah, Yemen, 2021.

Variable	Number^1^
**Households**	1657
**Household size**	6 (4, 8)^1^
**Households of Internally Displaced People**	593 (35·8%)
**District**	
** Khawkhah**	585 (35·3%)
** Tuhaytah**	364 (22·0%)
** Haiz**	338 (20·4%)
** Durayhimi**	340 (20·5%)
** Bayt Al Faqih**	36 (2·2%)
**Individuals**	10,360
**Age, years**	16 (6, 30) ^1^
**Female**	5,177 (50%)
**Pregnant and lactating women**	961 (9·3%)
**Joint members**	214 (2·1%)
**Absent (died/left) members**	77 (0·7%)

^1^Median (IQR) or Frequency (%).

### Children 6–59 months nutritional status and morbidity

**Fig 2:**
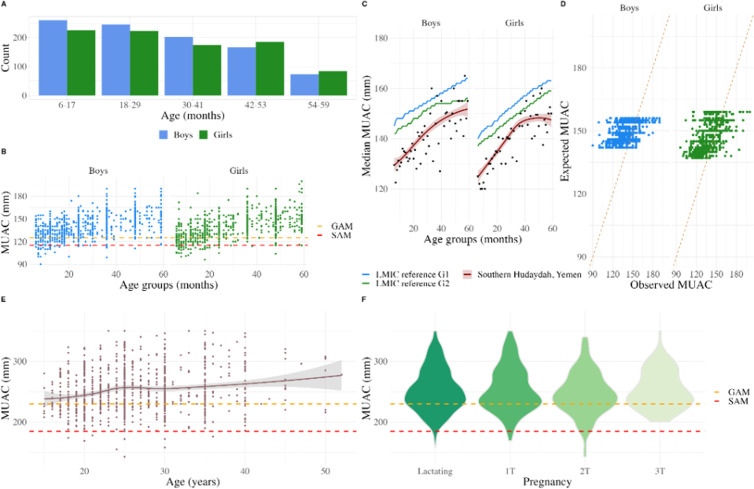
(A) Distribution of age (months) and sex of children aged 6 to 59 months, Southern Hudaydah, Yemen 2021. (B) MUAC (mm) measurements from sampled children aged 6-59 months, by sex, Southern Hudaydah, Yemen 2021. Dashed coloured lines refer to applied MUAC cutpoints to determine GAM (125mm - orange) or SAM (115mm - red). (C) Median MUAC (mm) by age and sex, among two groups (G1/G2) of 22 Low- and Middle-Income countries (LMIC) compared with sampled children aged 6-59 months (red), Southern Hudaydah, Yemen 2021. (D) Expected and observed MUAC (mm) of children aged 6 to 59 months, by sex, based on LMIC reference G2, Southern Hudaydah, Yemen 2021. (E) Distribution of MUAC (mm) measurements by age (years), Southern Hudaydah, Yemen 2021. Dashed coloured lines refer to MUAC cutpoints to determine GAM (230mm - orange) or SAM (183mm - red). (F) Violin plot of MUAC (mm) by pregnancy status, Southern Hudaydah, Yemen 2021. Legend. 1T: first trimester; 2T second trimester, 3T: third trimester.

In this survey, 1,760 children aged 6–59 months from 40 villages were assessed through MUAC (S4 File), including 909 boys and 851 girls (Fig 2A). The sex ratio was 1.06 (range of 0.87 to 1.16 between age groups), thus boys and girls were equally represented in our sample. The prevalence of GAM was 14.1% (95%CI: 12.2-16.2), with 4.0% (95%CI: 3.3-4.9) having SAM. We classified one child as having SAM presenting with bilateral pitting oedema. Table 2 presents children’s demographic and health characteristics by nutritional status (GAM and normal). We observed differences among girls and boys, girls having GAM prevalence of 16.2% (95%CI: 13.5-19.4), while boys had 12.1% (95%CI: 9.8-14.8) (p-value 0.013). Children experiencing GAM were significantly younger, with a median age of 12 months (IQR 8, 19) than those with normal nutritional status (median 30 months of age, p-value <0.001). Children aged 6–17 months had the highest prevalence, with 34.6% GAM (95%CI: 30.3-39.0), 23.2% of MAM (95%CI: 19.5-27.3) and 11.4% of SAM (95%CI: 8.7-14.7) (Fig 2B). We compared individual-level MUAC values against expected values by age and sex from non-malnourished children from 22 LMIC (Fig 2C) and found our study participants had a mean 8.3 mm reduced MUAC (8.8mm for the males and 7.7mm for the females) (Fig 2D). Overall, 73.8% (95%CI: 64.3-81.4) of the children had a lower MUAC than the expected MUAC for non-malnourished children of the same age and sex, 24.1% (95%CI: 16.7-33.4) were lower than two standard deviations (SD) and 7.9% (95%CI: 4.0-15.1) were lower more than three SD of the reference population.

**Table 2:** Demographic and health characteristics among children aged 6-59 months, by nutritional status, based on MUAC (mm) cutpoints and/or oedema, and estimated Odds Ratios and 95%Confidence Intervals for Global Acute Malnutrition, Southern Hudaydah, Yemen 2021.

Variable	Overall, N = 1,760^1^	GAM (MUAC < 125 mm), N = 248^1^	Normal (MUAC >= 125 mm), N = 1,512^1^	Univariate model, Unadjusted Odds Ratio (95% CI)	Full model, Adjusted^2^ Odds Ratio (95% CI)
**Age, months**	27 (16, 44)	12 (8, 19)	30 (18, 48)	0.92 (0.91-0.93)	0.92 (0.91-0.93)
**Girl**	851 (48%)	138 (56%)	713 (47%)	1.41 (1.07-1.84)	1.57 (1.18-2.11)
**Birthplace**					
Home	1,509 (86%)	218 (88%)	1,291 (85%)	1.0 (ref)	
Hospital	199 (11%)	24 (9.7%)	175 (12%)	0.81 (0.51-1.25)	
Private health centre	52 (3.0%)	6 (2.4%)	46 (3.0%)	0.77 (0.29-1.70)	
**Following nutrition program**	142 (57%)	142 (57%)	0 (0%)	–	–
**Reported sickness in the last 14 days**	958 (54%)	180 (73%)	778 (51%)	2.5 (1.87-3.38)	1.73 (1.27-2.39)

^1^Median (IQR) or Frequency (%), ^2^ Adjusted for age, sex and reported sickness in the last 14 days; Abbreviation: CI, confidence interval; ref, reference; MUAC, middle upper-arm circumference; GAM, global acute malnutrition.

To predict the effect of demographic and health factors on presenting GAM, we fitted fixed and mixed effects logistic regression models to our data. The most parsimonious model was a fixed effects logistic model using age, sex and reported sickness in the last 14 days (Fig 2D). A mixed model including cluster village as a random effect did not significantly improve the model performance (adjusted ICC 0.018). Being a child of an older age (OR 0.92, 95%CI: 0.91-0.93) was negatively associated with having GAM, but a child reported sickness in the last 14 days was 1.73 more likely to have GAM (95%CI: 1.27-2.39) (Table 2). A girl was 1.52 times more likely to present GAM (95%CI: 1.18-2.11). The VIF values for age, female sex, and reported sickness in the last 14 days were 1.015, 1.003, and 1.013, respectively. All VIF values were below 5, indicating no significant multicollinearity among these covariates.

Children’s birthplace was mostly at home (86%) and was not associated with differences between malnourished and normal nutritional status (Table 2). Although most children with GAM were already registered in a nutrition program, 43% did not have a specific nutrition follow-up. All GAM cases were offered a referral if not included yet in a nutrition program (n = 106) or presenting an acute medical complication. The referral was to the nearest inpatient therapeutic feeding centre (ITFC). During this assessment, 28 children with SAM presented health complications and 85% accepted being referred to the nearest ITFC. Of those, almost two thirds were admitted for medical treatment. More than half (n = 958, 54%) of the children assessed reported sickness in the last 14 days. Reported illnesses showed that respiratory diseases were the most prevalent, accounting for 33% (371 cases) of the total, followed closely by malaria at 31% (357 cases). Diarrhoea comprised 19% (213 cases). Meningitis symptoms, suspected measles, and road accidents each represent a smaller proportion of cases, at 0.5% (6 cases), 0.2% (2 cases), and 0.2% (2 cases), respectively. Reported sickness in the last 14 days was more prevalent among children with GAM compared to those without (73% vs 51%; p-value <0.001). Most of the children who reported sickness sought healthcare during that period (84%, 806/958). This proportion was higher in children with GAM (88%, 158/180). Among all children who sought healthcare, 63% (n = 508/806) paid out of pocket, and 76% (n = 611/806) had expenditure with medication. Regarding healthcare providers sought, the majority went to a public primary health unit (44%, n = 352/806), followed by a private primary health unit (28%, n = 222/806), almost a quarter went to a hospital (24%, 194/806) and 2% sought a traditional healer (n = 16/806).

### Maternal nutritional status

In our study, 961 women with a median age of 25 years (IQR 21, 30) were pregnant or lactating. Of these, 1.1% (95% CI: 0.6-1.9) had a MUAC <185 mm, classified as SAM and 24.7% (95%CI: 21.9-27.5) were classified as MAM. We observed a higher level of malnutrition for PLW below 30 years of age (p-value <0.001) (Fig 2E). Prevalence of GAM among PLW was 25.8% (95%CI: 23.0-28.6). MUAC measures according to pregnancy trimester or lactating status showed no statistical differences (Fig 2F). Regarding medical assistance received in the last 30 days, 155 PLW (17%) reported receiving it, including 40 out of 243 (16%) PLW with GAM.

### Prevalence of food insecurity

**Fig 3:**
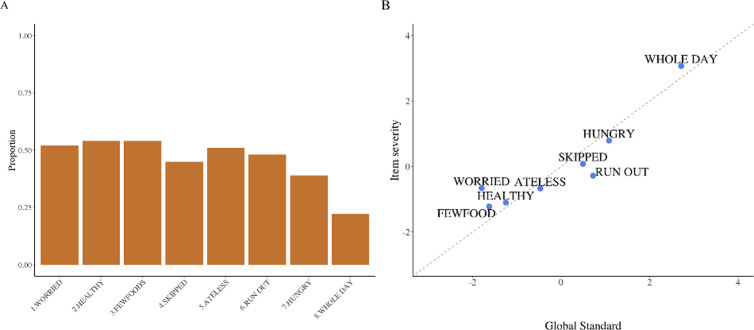
(A) Proportion of each food experience item, Southern Hudaydah, Yemen 2021. Orange bars represent the number of households with affirmative answers to each one of the eight FIES questions. (B) FIES global standard scale (x-axis) and item severity after calibration (y-axis) in Southern Hudaydah, Yemen 2021.

We used the data collected through the FIES to estimate the prevalence of food insecurity at internationally comparable levels of severity. The estimated prevalence of food insecurity was 54% (95%CI: 44–63) of households in Southern Hudaydah, with more than a fifth (22%, 95%CI: 15–31) being severely food insecure. The FIES translates a relative increasing severity of food experiences. The order of the food items (questions) along the scale among households in Southern Hudaydah was different from the standard administrative order (Fig 3A). When a household worries about food (*WORRIED*, question 1), they already did not have enough food to eat (*FEWFOOD*, question 3), were eating less healthy food (*HEALTHY*, question 2) and have already restricted their diet to eat less (*ATELESS*, question 5), still with the same number of meals (*SKIPPED*, question 4) (S5 File). The distance of the item parameters showed that households who experience severe food insecurity are more likely to go from hunger (*HUNGER,* question 7) to having no food for the whole day (*WHOLEDAY*) than their global counterparts (Fig 3B). We tested the data for statistical validation of the model assumptions, and it met acceptable statistical results (S5 File). We found that all eight items showed an adequate fit and discriminated equally well among respondents. However, the *WHOLEDAY* item was sensitive to outliers with unusual responses. The overall and mean “flat” Rasch reliability was 0.72 (minimum acceptable > 0.7) and the residual correlations were low (max.|0.29|).

### Mortality

During the recall period (three months), 23 people from the population surveyed died. The mean age of people who died was 30 years (range 0–90). The cause of death was unknown for more than half of the deceased, and the place of death was mostly at home (S6 File). Seven were children under 5 years of age and 65% were male. The majority sought healthcare (19/23), 12 (63%) went to a public hospital, 3 (16%) to a primary health centre, 2 (11%) to a private healthcare institution, 1 (5%) to a pharmacy and 1 (5%) to both hospital and private healthcare institution. These findings translate to a crude death rate (CDR) of 0.25 (95%CI: 0.15-0.42, design effect 1.44) per 10,000 individuals per day and the zero to five death rate (0–5DR) was 0.49 (95%CI: 0.20-1.19, design effect 1.75) per 10,000 children per day.

## Discussion

This study conducted in February-March 2021 in Southern Hudaydah showed that the health situation of the population in this region was alarming. Acute malnutrition and food insecurity estimates were above emergency thresholds, despite substantial current emergency interventions and programs [25].

International organisations have established levels for severity of acute malnutrition based on a threshold of prevalence [26,27]. When the prevalence of acute malnutrition exceeds 10% it is called an emergency, and requires appropriate treatment programs, including community and inpatient nutrition-specific interventions [28]. Our results showed a GAM of 14.1% among children aged 6–59 months, which means that a food emergency was occurring. The ITFC admission data showed that the survey led to an increased inpatient admissions due to the MSF referral, highlighting the lack of an effective case finding system. Children between the age of 6–17 months accounted for 34.6% of GAM and 11.4% of SAM in this study. Although the food insecurity levels and overall crises factors are a likely explanation for these results, this age group is also particularly vulnerable due to weaning and diet changes (from exclusive breastfeeding to food introduction). Nevertheless, even in 2013, two years before the actual conflict started, according to the Yemen National Health and Demographic Survey (YNHDS), only 15% of Yemeni children aged 6–23 months were fed in accordance with all three infant and young child feeding practices [29]. We also found sex differences in children with GAM, with an increased prevalence among girls. Undernutrition measured by anthropometric approaches is usually higher among boys [30]. A higher prevalence among girls in our survey could be due to overestimation of MUAC among boys, since we use the same cut-off point for both boys and girls, and there are body composition differences that could influence the accuracy. However, this could also be due to gender perceptions and factors related to differential feeding practices for boys and girls [31]. A year later, 2022, data from the Yemen Nutrition Cluster showed a children’s GAM of 26.3% (SAM 7.0%), despite the current interventions [32]. We also compared individual MUAC measurements to existing age- and sex-specific estimates from non-malnourished populations in 22 different LMIC. We found that the applied single cut-off point used in emergencies underestimated the prevalence of GAM when compared to age- and sex-specific references from 22 LMIC (14.1% vs 24.1%) and the prevalence of SAM (4.0 vs 7.9%). The WHO also developed reference growth standards for infants and young children on a community-based, multi-country project (Brazil, Ghana, India, Norway, Oman, and the United States) [33]. Because WHO child growth references come from both high-income and LMIC, and from an overall lower number of countries, the developed 22 LMIC reference provides a more realistic comparison for Yemen children.

Prevalence of malnutrition among PLW was also alarming in our study and, although risk factors for this estimate were not assessed here, food insecurity, frequent childbearing (family size 6–8) and prioritisation for children food could be likely risk factors [34,35]. Assessing results one year later than this study, in 2022 PLW’s GAM was 32.1% in the same districts, despite efforts taken [32]. We used MUAC to assess PLW nutritional status. Adult malnutrition is usually measured by calculating Body Mass Index (BMI). However, indices including body weight, such as BMI, are not recommended for PLW, since weight changes with the growing foetus. On the other hand, MUAC does not change significantly through pregnancy, and it is strongly associated with adverse health outcomes (e.g., low birth weight), therefore, it is the preferred nutritional index for PLW in the African and Asian contexts [36,37].

As of 2021, more than half of the Southern Hudaydah population was found to be food insecure in the last 12 months and more than a fifth were severely food insecure. This can be translated to 127,446 individuals’ food insecure in Southern Hudaydah in Feb-March 2021, 75,522 moderately insecure and 51,922 severely food insecure. The order of the food items, and so the severity of food experiences across the Southern Hudaydah population was experienced differently from the one gathered from global results. Responses showed that by the time the Southern Hudaydah population worried they would not have enough food to eat, they had already restricted their meals to a limited number of food items and begun to limit their intake of vegetables and fruits. Fundamentally, this indicates that relative severity is not fixed across countries and several factors could explain it, such as cultural habits, but also perception of the question which involves English-Arabic language translation efforts. The Sustainable Development Goals (SDG) are a universal framework to guide countries improving health, education, economy, equity, justice, and other fundamental goals of a prosperous population [15]. The SDG 2 targets *Zero Hunger*, and specifically *Target 2.1* populations in vulnerable situations, ensuring access to safe and enough food [38]. Although our survey does not allow for the assessment of causal pathways for these findings, food insecurity was alarming and likely contributed as one of the main causes for the nutrition crises in this population. These results are similar to results found in other humanitarian crises where FIES was used [39,40]. Our validation analysis was consistent with assumptions used in the model and showed that FIES was a valid measurement tool for the Southern Hudaydah population in the survey period.

High death rates normally indicate that there is a health problem and are particularly worrying when prevalence of malnutrition is high. The retrospective mortality results showed a CDR and 0–5DR below the UN High Commissioner for Refugees alert threshold (1 and 2 deaths/10,000 people/day, for CDR and 0–5DR respectively) [41]. We found the low CDR and 0–5DR to be a close reflection of the situation due to the rigorous survey quality control, including having done multiple trainings, and piloting of the survey in all districts. Nevertheless, the primary scope of this survey was the nutrition and food insecurity levels and, therefore, the less time dedicated to these questions might have biassed the death rates results [13]. Nevertheless, non-food risk factors for malnutrition and mortality and the protracted nature of the crisis could worsen the situation at any point, even if rates were not alarming at the time of the study [42]. It is possible that death rates would increase with higher levels of malnutrition and morbidity, however, it could also rise in the setting of relatively low prevalence of acute malnutrition and acute malnutrition may rise without substantial increases in death rates.

Around the world, leading causes for children’s morbidity and mortality are all communicable diseases, being acute respiratory diseases, diarrhoeal diseases, and malaria the most common [43]. In the 14 days before the interview, more than half of the children under 5 were reported to be sick. Assessing the proportions by groups of diseases, those from the YNHDS were lower in comparison to this assessment, except for diarrhoeal disease. Acute respiratory disease was reported by 33% of the sick children (12% in YNHDS), 31% reported malaria (32% reporting fever in YNHDS, as a proxy for malaria) and 19% reported diarrhoeal disease (31% in YNHDS) [29]. The great majority of the households reporting a child sickness in the last 14 days sought healthcare and paid for it and/or medication. Due to the current economic situation of Yemen, these results might represent a substantial burden in a household budget, composed of a median of 6 members, 18% children under five, and of those, half reporting sickness in the last two weeks.

Our study has several limitations. First, the cross-sectional nature of our study only assessed acute malnutrition, food insecurity and morbidity point estimates and, consequently, cannot indicate causal explanations for the findings. Although the population sample is representative of the Southern Hudaydah region, these findings cannot be used to draw conclusions about health-related outcomes at the district level. In the same way, the unique context of this region, including its proximity to active frontlines, limits the generalizability of our findings to other areas of Yemen where conditions differ. That said, factors contributing to malnutrition - such as conflict, limited food access, and disrupted healthcare - are challenges shared by other regions of the country. The reliance on population data provided by the administrative authorities, which, while the most reliable source available, may not fully capture recent demographic changes in a poorly resourced and volatile setting. Despite having different teams in each district conducting interviews and measurements, we conducted a refreshment training prior to the start of the study and ran a pilot day for each one of them. Nevertheless, the overall data quality of our study was scored as acceptable. The gold-standard method to assess GAM among children through household surveys is based on the weight-for-height score and/or presence of bilateral oedema. Due to the high-risk setting and rapid scope of our assessment, GAM was based on MUAC measurements [41,44], though still being a beneficial measurement specially on identifying children with a high-risk of death [45]. The recall period used in this study was 96 days, falling within the recommended duration in household surveys (three to six months) [13]. Calendar dates of important local events were used when asking about deceased family members. Our questionnaire was validated through field pilot tests and a two-step translation approach was adopted to decrease semantic errors (English-Arabic-English). However, due to the conflict setting, we cannot exclude the presence of recall biases. The period of data collection also might have impacted the estimated mortality rate, due to more/less active violent periods. We also cannot exclude misclassification biases on the morbidity results. This data was collected based on the caregivers’ perception of their child being ill, without further validation by a medical professional or access to clinical information/records for the child. For the food insecurity estimations, we used a cross-validation process of the Rasch model assumptions. Equal discrimination and conditional independence between the food item questions was met, nevertheless *WHOLEDAY* item was sensitive to outliers with unusual responses. Reasons for these differences could be due to question formulation and translation as discussed above. Due to the high-risk setting of this study and to keep data collection times as short as possible at both household and village levels, we did not inquire about detailed common food habits or other characteristics relevant to nutrition, such as for micronutrients deficiencies or specific diets.

Our results showed lower but above the threshold levels of acute malnutrition and food insecurity when compared to other sources in the same region^6^. These results favoured multisectoral nutrition interventions for women and children in Yemen as described in the UN Global Action Plan on Child Wasting. Due to the situation of high food insecurity, interventions should ensure access to food and adequate nutrition, but also unrestricted access to humanitarian relief. Increasing numbers of children and PLW diagnosed and under treatment could be achieved by community-based programs and simplified approaches, such as MUAC measurement training by the caregiver/family and treatment by community health workers [46–48] .Nevertheless, the underlying causes for undernutrition are complex and interconnected [10]. Even with appropriate shelter, settlement, healthcare and drinking-water, sanitation and hygiene (WASH) responses, efforts to increase livelihood opportunities will not be enough when the most important basic cause is not solved. Full access humanitarian assistance, de-escalation and peace initiatives will certainly protect health and reinstate livelihoods, specifically among the most vulnerable groups.

### Interpretation

This representative survey found an alarming health situation, with acute malnutrition above emergency thresholds, and more than half of the households being food insecure.

## Supporting information

S1 File Sampling design and calculations. (DOCX)

S2 File Food Insecurity Experience Scale (FIES) questions. (DOCX)

S3 File Death rates estimate calculations. (DOCX)

S4 File Mean MUAC (mm) and standard deviation (SD) interval of under five years of age children by cluster. (DOCX)

S5 File Food insecurity: statistical validation and equating. (DOCX)

S6 File Main causes of death by place of death. (DOCX)

S1 Checklist Inclusivity in global research. (PDF)
