# Supplementary material for: Acute malnutrition and food insecurity in Yemen, 2021: Evidence from a two-stage cluster randomised survey in a protracted crisis
Source: PLOS Glob Public Health. 2025 Jul 11;5(7):e0004331. doi: 10.1371/journal.pgph.0004331 (PMC12250524; doi:10.1371/journal.pgph.0004331)
Supplement: S1 File — (DOCX) [file pgph.0004331.s001.docx]

**S1. Sampling design and calculations**

**Sampling design**

Sample size was calculated for each study objective. We used the Emergency Nutrition Assessment (ENA) for SMART software, updated version of January 2020 for these calculations. The sample size of the assessment was determined by the higher sample size.[^1^](https://paperpile.com/c/TwMq6V/QFpK)

*First-stage sampling – cluster selection*

A cross sectional, standard two-stage cluster sampling method was used to randomly select clusters from the primary sampling units in the first stage. The primary sampling units were villages, whereas the second sampling units were households. The ENA for SMART software of January 2020 was used to determine the cluster assignment using the updated village‐level population data. We used a nominal list of villages provided by the District Health Authorities (Supplementary Table 1). The highest sample size for the assessment’s objectives was 1472 households. Therefore, a sampling plan of 40 clusters was surveyed to obtain the total number of 1480 households, each cluster with 37 households. We used the SMART software to randomly assign clusters, with the chance of each village being chosen proportional to its population size[^1^](https://paperpile.com/c/TwMq6V/QFpK).

*Second-stage sampling - household selection*

At the second stage, random sampling of households was carried out in the selected clusters. Due to operational challenges and difficulty to assess and update maps of selected communities (new locations due to internal displaced people), a geographical information systems approach was not feasible. We used “the pen method”, by throwing a pen on the ground in the central point of the cluster (village recognised centre), and a line drawn from the direction it pointed towards to the edge of the cluster[^2^](https://paperpile.com/c/TwMq6V/Hfxy). To prevent centre-bias, the team walked in the direction of the pen until the edge of the cluster was reached, then threw the pen again and households along this line were counted until the edge of the cluster was reached. One of these was selected using a random number table as the first to be interviewed in the cluster. The next household following in order of physical proximity was then interviewed, until the desired cluster of 37 households was completed. Physical proximity was defined as being the front door of a household closest to the front door of the household that was just interviewed. If more than one house could be selected, the house to the left as one stands looking out of the doorway of the household just interviewed, was chosen.

**Supplementary Table 1.** List of the 40 assigned clusters of the assessment, including the correspondent population size

| Cluster | Population size (n) |
| --- | --- |
| 1 | 358 |
| 2 | 7246 |
| 3 | 1967 |
| 4 | 5010 |
| 5 | 2441 |
| 6 | 1348 |
| 7 | 2004 |
| 8 | 1623 |
| 9 | 774 |
| 10 | 912 |
| 11 | 2340 |
| 12 | 3030 |
| 13 | 540 |
| 14 | 2370 |
| 15 | 984 |
| 16 | 1242 |
| 17 | 3608 |
| 18 | 2490 |
| 19,20 | 12124 |
| 21 | 2465 |
| 22 | 2029 |
| 23 | 2284 |
| 24 | 1016 |
| 25,26 | 6132 |
| 27 | 600 |
| 28 | 596 |
| 29 | 1055 |
| 30 | 1260 |
| 31, 32, 33, 34, 35 | 25082 |
| 36 | 5493 |
| 37 | 5379 |
| 38, 39 | 10734 |
| 40 | 456 |
| Reserve cluster | 934 |
| Reserve cluster | 1458 |
| Reserve cluster | 5556 |
| Reserve cluster | 1280 |
| Reserve cluster | 2752 |

The sample size calculations and assumptions to meet the study objectives were calculated as follows:

**Supplementary Table 2.** Sample size assumptions for estimating the prevalence of acute malnutrition among children aged 6-59 months of age in Southern Hudaydah Governorate, Yemen

| Parameters | Value | Assumptions based on context |
| --- | --- | --- |
| Estimated prevalence of GAM (%) | 27 | According to the IPC Analysis - Acute Malnutrition January - July 2020 and Projection for August - December 2020 [^3^](https://paperpile.com/c/TwMq6V/BfKi9) |
| Desired precision (%) | 5 | Since the GAM prevalence is high, a precision of 5% is appropriate[^1^](https://paperpile.com/c/TwMq6V/QFpK) |
| Design effect | 2 | To compensate for possible heterogeneity between clusters[^4^](https://paperpile.com/c/TwMq6V/UCmAg) |
| Household size (n) | 6 | Source: South Hudaydah governorate health authorities |
| Children under-five (%) | 18 | Source: South Hudaydah governorate health authorities |
| Non-respondents (%) | 10% | Standard estimate [^1^](https://paperpile.com/c/TwMq6V/QFpK) |
| Children to be included (n) | 659 |  |
| Households to be included (n) | 754 |  |

**Supplementary Table 3.** Sample size assumptions for estimating the prevalence of acute malnutrition among PLW in Southern Hudaydah Governorate, Yemen

| Parameters | Value | Assumptions based on context |
| --- | --- | --- |
| Estimated prevalence of GAM (%) | 31 | According to the IPC Analysis - Acute Malnutrition January - July 2020 and Projection for August - December 2020[^3^](https://paperpile.com/c/TwMq6V/BfKi9) |
| Desired precision (%) | 5 | Since the GAM prevalence is high, a precision of 5% is appropriate[^1^](https://paperpile.com/c/TwMq6V/QFpK) |
| Design effect | 2 | To compensate for possible heterogeneity between clusters[^4^](https://paperpile.com/c/TwMq6V/UCmAg) |
| Household size (n) | 6 | Source: South Hudaydah governorate health authorities |
| Pregnant and lactating women (%) | 10 | Source: South Hudaydah governorate health authorities |
| Non-respondents (%) | 10% | Standard estimate [^1^](https://paperpile.com/c/TwMq6V/QFpK) |
| PLW to be included (n) | 716 |  |
| Households to be included (n) | 1472 |  |

**Supplementary Table 4.** Sample size assumptions for estimating the U5MR in Southern Hudaydah Governorate, Yemen

| Parameters | Values |
| --- | --- |
| Estimated U5 MR (per 10,000/day) | 1.94* |
| Precision (per 10,000/day) | 0.6 |
| Design Effect | 2.0[^1^](https://paperpile.com/c/TwMq6V/QFpK) |
| Household Size (n) | 6[^5^](https://paperpile.com/c/TwMq6V/RAzhX) |
| Recall Period (days) | 90[^6^](https://paperpile.com/c/TwMq6V/jdR8f) |
| Non-respondents (%) | 10[^1^](https://paperpile.com/c/TwMq6V/QFpK) |
| Children to be included (n) | 5008 |
| Households to be included (n) | 927 |

*Sample size was calculated for all five districts of Southern Hudaydah Governorate (Table 4). Since previous estimates on U5MR are different within the five districts, Al Khawkha (1.2 per 10,000/day), Al Tuhayat (1.76 per 10,000/day), Bayt Al-Faqiah (1.94 per 10,000/day), Haiz (1.7 per 10,000/day) and Al Durayhami (1.41 per 10,000/day), we adopted the most parsimonious value of 1.94 per 10,000/day.

**Supplementary Table 5.** Sample size assumptions for estimating the CMR in Southern Hudaydah Governorate, Yemen

| Parameters | Values |
| --- | --- |
| Estimated CMR 2018 (per 10,000/day) | 0.16[^7^](https://paperpile.com/c/TwMq6V/09BnC) |
| Precision (per 10,000/day) | 0.3 |
| Design Effect | 2.0[^1^](https://paperpile.com/c/TwMq6V/QFpK) |
| Household Size (n) | 6[^5^](https://paperpile.com/c/TwMq6V/RAzhX) |
| Recall Period (days) | 90[^6^](https://paperpile.com/c/TwMq6V/jdR8f) |
| Non-respondents (%) | 10[^1^](https://paperpile.com/c/TwMq6V/QFpK) |
| People to be included (n) | 1652 |
| Households to be included (n) | 306 |

**References**

1 [Methodology S. Sampling methods and sample size calculation for SMART methodology. 2012.](http://paperpile.com/b/TwMq6V/QFpK)

2 [Turner AG. Sampling frames and master samples. *United Nations secretariat statistics division* 2003;1–26.](http://paperpile.com/b/TwMq6V/Hfxy)

3 [Yemen: IPC acute Food insecurity analysis - January - December 2020 (issued October 2020). ReliefWeb.](http://paperpile.com/b/TwMq6V/BfKi9) <https://reliefweb.int/report/yemen/yemen-ipc-acute-food-insecurity-analysis-january-december-2020-issued-october-2020> [(accessed Jan 22, 2024).](http://paperpile.com/b/TwMq6V/BfKi9)

4 [World Health Organization. Regional Office for the Eastern Mediterranean. Field Guide on Rapid Nutritional Assessment in Emergencies. World Health Organization, Regional Office for the Eastern Mediterranean, 1995.](http://paperpile.com/b/TwMq6V/UCmAg)

5 [Mophp P, Yemen. Yemen national health and demographic survey 2013. 2015; published online July 1.](http://paperpile.com/b/TwMq6V/RAzhX) <https://dhsprogram.com/publications/publication-fr296-dhs-final-reports.cfm>[.](http://paperpile.com/b/TwMq6V/RAzhX)

6 [Checchi F. Estimation of population mortality in crisis-affected populations: guidance for humanitarian coordination mechanisms. Geneva: World Health Organization; 2018. .](http://paperpile.com/b/TwMq6V/jdR8f)

7 [Death rate, crude (per 1,000 people) - Yemen, Rep. World Bank Open Data.](http://paperpile.com/b/TwMq6V/09BnC) <https://data.worldbank.org/indicator/SP.DYN.CDRT.IN?locations=YE> [(accessed Feb 5, 2024).](http://paperpile.com/b/TwMq6V/09BnC)
