# Supplementary material for: Acute malnutrition and food insecurity in Yemen, 2021: Evidence from a two-stage cluster randomised survey in a protracted crisis
Source: PLOS Glob Public Health. 2025 Jul 11;5(7):e0004331. doi: 10.1371/journal.pgph.0004331 (PMC12250524; doi:10.1371/journal.pgph.0004331)
Supplement: S2 File — (DOCX) [file pgph.0004331.s002.docx]

# **S2. Food Insecurity Experience Scale (FIES) questions**

The FIES questions[^8^](https://paperpile.com/c/TwMq6V/OnPwf) in our survey refer to the self-reported food related behaviours of each household. The questions are designed as follows:

*During the last 12 months, was there a time when, because of lack of money or other resources:*

1. *You were worried you would not have enough food to eat? (WORRIED)*
2. *You were unable to eat healthy and nutritious food? (*HEALTHY)
3. *You ate only a few kinds of foods? (FEW FOODS)*
4. *You had to skip a meal? (SKIPPED)*
5. *You ate less than you thought you should? (ATELESS)*
6. *Your household ran out of food? (RUN OUT)*
7. *You were hungry but did not eat? (HUNGRY)*
8. *You went without eating for a whole day? (*WHOLE DAY*)*
