# Supplementary material for: Acute malnutrition and food insecurity in Yemen, 2021: Evidence from a two-stage cluster randomised survey in a protracted crisis
Source: PLOS Glob Public Health. 2025 Jul 11;5(7):e0004331. doi: 10.1371/journal.pgph.0004331 (PMC12250524; doi:10.1371/journal.pgph.0004331)
Supplement: S3 File — (DOCX) [file pgph.0004331.s003.docx]

**S3. Death rates estimate calculations**

We calculated the crude death rate, expressed as deaths per 10,000 individuals per day, using the following formula[^6^](https://paperpile.com/c/TwMq6V/jdR8f):

CDR = number of deaths among total study’s participants/ total study’s participants at mid-point in the recall period /10,000 *recall period.

The zero-to-five death rate (0-5DR), expressed as deaths per 10,000 children from zero to five years of age per day, was calculated using the formula[^6^](https://paperpile.com/c/TwMq6V/jdR8f):

0-5DR = number of deaths among study’s participants aged zero to five years/ study’s participants aged zero to five years at mid-point in the recall period /10,000 *recall period

The denominators used to estimate crude and age-specific death rates were the average study’s population size at the end of the period minus half of those joining the sample during the recall period (new-borns and new household members) plus half of those leaving the sample during the recall period (death, travel, or other permanent absence).
