# Supplementary material for: Acute malnutrition and food insecurity in Yemen, 2021: Evidence from a two-stage cluster randomised survey in a protracted crisis
Source: PLOS Glob Public Health. 2025 Jul 11;5(7):e0004331. doi: 10.1371/journal.pgph.0004331 (PMC12250524; doi:10.1371/journal.pgph.0004331)
Supplement: S4 File — (DOCX) [file pgph.0004331.s004.docx]

**S4. Mean MUAC (mm) and standard deviation (SD) interval of under five years of age children by cluster, Southern Hudaydah, Yemen 2021**

| Cluster | Number of children under five years of age | Mean MUAC in mm ± SD |
| --- | --- | --- |
| Cluster 1 | 40 | 139,10 ±15,07 |
| Cluster 2 | 50 | 141,00 ±14,03 |
| Cluster 3 | 45 | 138,50 ±15,51 |
| Cluster 4 | 66 | 140,10 ±14,05 |
| Cluster 5 | 36 | 131,70 ±13,66 |
| Cluster 6 | 27 | 138,30 ±12,50 |
| Cluster 7 | 52 | 132,70 ±12,49 |
| Cluster 8 | 46 | 140,50 ±12,62 |
| Cluster 9 | 40 | 138,10 ±12,09 |
| Cluster 10 | 22 | 135,60 ±15,71 |
| Cluster 11 | 47 | 141,70 ±16,67 |
| Cluster 12 | 59 | 146,60 ±14,15 |
| Cluster 13 | 33 | 139,80 ±14,28 |
| Cluster 14 | 28 | 140,70 ±14,18 |
| Cluster 15 | 47 | 139,30 ±16,88 |
| Cluster 16 | 39 | 141,60 ±14,57 |
| Cluster 17 | 54 | 141,10 ±19,07 |
| Cluster 18 | 54 | 138,20 ±15,35 |
| Cluster 19 | 34 | 137,40 ±15,44 |
| Cluster 20 | 62 | 143,60 ±26,19 |
| Cluster 21 | 49 | 145,20 ±18,32 |
| Cluster 22 | 56 | 140,20 ±15,51 |
| Cluster 23 | 34 | 136,90 ±17,06 |
| Cluster 24 | 41 | 137,20 ±13,86 |
| Cluster 25 | 49 | 140,90 ±16,04 |
| Cluster 26 | 58 | 138,00 ± 9,89 |
| Cluster 27 | 44 | 143,10 ±18,00 |
| Cluster 28 | 39 | 137,20 ±14,14 |
| Cluster 29 | 31 | 138,60 ±11,01 |
| Cluster 30 | 32 | 145,50 ±14,21 |
| Cluster 31 | 39 | 136,30 ±13,32 |
| Cluster 32 | 33 | 139,40 ±20,31 |
| Cluster 33 | 32 | 149,40 ±16,66 |
| Cluster 34 | 34 | 143,20 ±13,70 |
| Cluster 35 | 31 | 138,10 ±12,57 |
| Cluster 36 | 88 | 148,50 ±21,57 |
| Cluster 37 | 44 | 140,90 ±14,90 |
| Cluster 38 | 61 | 139,00 ±15,86 |
| Cluster 39 | 43 | 144,70 ±15,69 |
| Cluster 40 | 31 | 137,40 ±14,88 |
