# Supplementary material for: Acute malnutrition and food insecurity in Yemen, 2021: Evidence from a two-stage cluster randomised survey in a protracted crisis
Source: PLOS Glob Public Health. 2025 Jul 11;5(7):e0004331. doi: 10.1371/journal.pgph.0004331 (PMC12250524; doi:10.1371/journal.pgph.0004331)
Supplement: S5 File — (DOCX) [file pgph.0004331.s005.docx]

**S5. Food insecurity: statistical validation and equation**

*Statistical validation*

We used statistical modelling of FIES data to produce estimates of the prevalence of food insecurity at comparable levels of severity.^1,^[^2^](https://paperpile.com/c/TwMq6V/jI0qn+OnPwf) The expected value of all infits is 1.0 and values between 0.7 and 1.3 are considered to have an acceptable fit consistent with the assumption of equal discrimination. Our FIES data met acceptable ranges for fit statistics for all eight items (range 0.84 to 1.18). Of the outfit statistics, one surpassed the acceptable maximum of 2, indicating that the *WHOLEDAY* item was sensitive to outliers with unusual responses. The overall and mean “flat” Rasch reliability was 0.72 and provides evidence that the scale effectively discriminates between respondents who have more or less food insecurity and reflects reasonably good model fit (minimum acceptable > 0.7). A residuals correlation between a pair of items is considered high if it is above |0.4|. The residual correlations were low (max.|0.29|) enough to infer that each item captures a unique aspect of food insecurity. The statistical validation results suggest that all items of FIES can be used for equation and are a valid measurement tool for the Southern Hudaydah.

**Supplementary Table 7.** Item parameters and fit statistics of FIES Scale, Southern Hudaydah, Yemen 2021

| Item | Severity | Standard error | Infit | Outfit | Complete non-extreme sample (n, %) |
| --- | --- | --- | --- | --- | --- |
| WORRIED | -0.658 | 0.110 | 1.113 | 1.059 | 467, 71.1 |
| HEALTHY | -1.105 | 0.115 | 1.179 | 1.209 | 508, 77.3 |
| FEWFOODS | -1.220 | 0.117 | 0.862 | 0.658 | 518, 78.8 |
| SKIPPED | 0.080 | 0.105 | 1.027 | 0.881 | 392, 59.7 |
| ATELESS | -0.669 | 0.110 | 0.855 | 1.015 | 468, 71.2 |
| RUNOUT | -0.284 | 0.107 | 0.838 | 0.709 | 430, 65.4 |
| HUNGRY | 0.790 | 0.103 | 0.872 | 0.731 | 313, 47.6 |
| WHOLEDAY | 3.065 | 0.142 | 1.141 | 2.186 | 59, 9.0 |

*Equating*

To compare the study’s FIES scale with the FIES global standard scale, we calibrated the two scales on a common metric by equating the mean and standard deviation. The resulting correlation between the FIES items was 91.%. By looking at table 8 we can see that the *RUNOUT* item had the highest absolute difference. After removing it from the equation, the correlation between other items increased and *WORRIED* showed the highest absolute difference. After removing it too, the correlation between common items was 98% and expressed a good equation scenario by the six remaining items.[^1,2^](https://paperpile.com/c/TwMq6V/jI0qn+OnPwf)

**Supplementary Table 8.** Absolute differences between study’s data and global standard item severity, Southern Hudaydah, Yemen 2021

| FIES items | Absolute difference between study’s data and standard item severities (after calibration) | Absolute difference between study’s data and standard item severities (after calibration and removing RUNOUT) | Absolute difference between study’s data and standard item severities (after calibration and removing RUNOUT and WORRIED) |
| --- | --- | --- | --- |
| WORRIED | 0.94 | 0.83 | 1.14 |
| HEALTHY | 0.00 | 0.12 | 0.14 |
| FEWFOOD | 0.23 | 0.11 | 0.40 |
| SKIPPED | 0.38 | 0.52 | 0.41 |
| ATELESS | 0.26 | 0.39 | 0.20 |
| RUNOUT | 0.95 | 1.09 | 1.00 |
| HUNGRY | 0.20 | 0.35 | 0.29 |
| WHLDAY | 0.61 | 0.44 | 0.35 |
| Correlation between common items (%) | 91.02 | 94.65 | 97.68 |

**References**

1 [Cafiero C, Nord M, Viviani S, *et al.* Voices of the hungry. *Methods for estimating comparable prevalence rates of food insecurity experienced by adults throughout the world Rome: FAO* 2016.](http://paperpile.com/b/TwMq6V/OnPwf) <https://www.fao.org/fileadmin/templates/ess/voh/Objectives_and_Challenges.pdf>[.](http://paperpile.com/b/TwMq6V/OnPwf)

2 [Cafiero C, Viviani S, Nord M. Food security measurement in a global context: The food insecurity experience scale. *Measurement* 2018; **116**: 146–52.](http://paperpile.com/b/TwMq6V/jI0qn)
