# Supplementary material for: Acute malnutrition and food insecurity in Yemen, 2021: Evidence from a two-stage cluster randomised survey in a protracted crisis
Source: PLOS Glob Public Health. 2025 Jul 11;5(7):e0004331. doi: 10.1371/journal.pgph.0004331 (PMC12250524; doi:10.1371/journal.pgph.0004331)
Supplement: S6 File — (DOCX) [file pgph.0004331.s006.docx]

**S6. Main causes of death by place of death, Southern Hudaydah, Yemen 2021**

| Main causes of death/  Place of death | Total  (N=23)^1^ | Hospital^1^ | Home^1^ | Way to Hospital^1^ | Other^1^ |
| --- | --- | --- | --- | --- | --- |
| Injury/traumatic | 3 (13,0) | 2 (8,7) | - | 1 (4.3) | - |
| Malaria | 2 (8.7) | 1 (4.3) | 1 (4.3) | - | - |
| Respiratory disease | 1 (4.3) | 1 (4.3) | - | - | - |
| Post-partum | 3 (13.0) | - | 2 (8.7) | 1 (4.3) | - |
| Diarrhoea/gastroenteritis | 2 (8.7) | 1 (4.3) | 1 (4.3) | - | - |
| Unknown | 12 (52.3) | 1 (4.3) | 8 (34.8) | - | 2 (8.7) |

^1^ Frequency (%)
